# Exploring the Value of Paired Microbiology and Histology in Chronic Osteomyelitis and Fracture-Related Infections

**DOI:** 10.3390/antibiotics14121277

**Published:** 2025-12-16

**Authors:** Anton A. N. Peterlin, Martin McNally, Nicole L. Henriksen, Sophie A. Blirup-Plum, Ann Jørgensen, Andreas Ibrahim Jørgensen, Inger Brock, Hans Gottlieb, Louise K. Jensen

**Affiliations:** 1 Department of Veterinary and Animal Sciences, University of Copenhagen, 1870 Frederiksberg, Denmark; anton.peterlin@sund.ku.dk (A.A.N.P.);; 2 Department of Orthopedic Surgery, Herlev Hospital, 2730 Herlev, Denmark; ann.joergensen.01@regionh.dk (A.J.);; 3 The Bone Infection Unit, Nuffield Orthopaedic Centre, Oxford University Hospitals, Oxford OX3 7HE, UK; 4 Department of Microbiology, Herlev Hospital, 2730 Herlev, Denmark

**Keywords:** fracture-related infections, chronic osteomyelitis, paired, histology, microbiology, preoperative antibiotics, outcome

## Abstract

**Background**: Microbiological culture and histology are gold standards for diagnosing chronic osteomyelitis (cOM) and fracture-related infection (FRI). This study investigated whether combining these modalities within a single tissue sample provides additional insight into disease severity. We hypothesized that high neutrophil and osteoclast numbers correlate with culture-positive microbiology and that double-positive samples may indicate more severe disease. **Methods**: In this prospective single-centre study, adults undergoing surgery for confirmed FRI or cOM were included. Clinical and disease classification data (FRI and BACH) were recorded. Five deep-tissue samples were collected intraoperatively and divided for paired microbiological culture and histological assessment of neutrophil infiltration, according to international diagnostic guidelines. **Results**: Forty-one patients were included (11 cOM, 30 FRI) of whom 68% received preoperative antibiotics. Nineteen patients (46%) were identified as culture-positive, while 32 patients (78%) were histologically positive according to international diagnostic guidelines, respectively. Among the 205 samples, 31% were culture-positive, 56% histology-positive, and 26% double-positive. Histological scores were significantly higher in culture-positive samples (*p* < 0.001). Treatment failure occurred in seven patients (18%), all with FRI. Paired positive samples were associated with increased odds of clinical failure and earlier revision, with odds increasing 1.68-fold for each additional paired positive sample (95% CI, 1.10–2.77). **Conclusions**: The paired analysis demonstrated a strong concordance between culture-positivity and suppurative inflammation within the same sample. Combining microbiology and histology may help identify patients at increased risk of revision and enhance diagnostic certainty, particularly in patients identified as culture-negative.

## 1. Introduction

Current recommendations for the management of chronic osteomyelitis (cOM) and fracture-related infections (FRIs) follow similar evidence-based protocols [1,2,3,4,5,6]. These include preoperative optimization with an antibiotic holiday of at least two weeks, if possible, obtaining at least five deep tissue samples for microbiology and two for histology, thorough debridement with hardware removal when indicated, dead space management, application of a local bioabsorbable antibiotic carrier, and a tailored postoperative antimicrobial regimen.

Microbiological culture and histology are among the diagnostic gold standards in cOM and FRI [1,7,8]. Cultures enable pathogen identification and guide postoperative antibiotic therapy; however, their reliability may be affected by prior antibiotic exposure. Although evidence is mixed, several reports suggest decreased diagnostic sensitivity in patients pretreated with antibiotics [9,10,11]. Histology offers strong discriminatory value when applied in a bimodal fashion, where the presence of at least five neutrophils (NPs) per high-power field (HPF) indicates infection with high specificity, and the complete absence of NPs strongly supports an aseptic process [8,12].

Diagnosing chronic bone infection remains challenging due to the difficulty of detecting bacteria within deep tissue samples [13,14,15]. Bacteria often exist in low numbers, in small aggregates, embedded in biofilms or within host cells, making culture results unreliable and sometimes falsely negative even in cases with clinically visible confirmatory infection criteria [16]. Histology is valuable in such cases by identifying neutrophilic infiltration indicative of infection [12]. However, low-grade or indolent infections may elicit only minimal inflammatory responses and thus escape detection by conventional histological criteria [17]. Thus, both the microbiological and histological diagnostic modalities have lower sensitivity compared to specificity. Furthermore, histology remains underutilized in clinical practice. Many orthopaedic centres do not routinely perform histological analysis in suspected bone infections due to the lack of standardized protocols [18]. Even in large multicentre studies, such as the OVIVA randomized controlled trial [19], histology is not obtained in approximately 40% of the enrolled patients.

This study investigated if pairing the diagnostic modalities of microbiology and histology, can provide new insights into bone infections. According to chemotaxis theory, neutrophil density should increase as the distance from bacterial colonies decreases [20]. Likewise, osteoclasts are expected to localize near bacteria, as infection-induced signals and bacterial factors drive their maturation and osteolytic activity [21,22,23,24,25]. Therefore, the hypothesis of the present study was that a high neutrophil and osteoclast count, respectively, within a cOM and FRI sample correlates with culture-positive microbiology, and that paired diagnostic positivity (culture-positive microbiology and ≥five NPs per high-power field) indicates a more severe disease severity driven by a highly activated innate immune response.

## 2. Results

### 2.1. Patient Demographics

In total, 41 patients were included, with 11 in the cOM group and 30 in the FRI group (Table 1). cOM patients were significantly older than those in the FRI group. No significant differences were observed for comorbidities such as, diabetes, heart failure, or COPD, respectively. Antibiotics were administered prior to index surgery (no cessation up to sampling) in 68.3% of the patients. Treatment failed with the need for revision surgery in seven patients (17.9%), all in the FRI group. 61% of patients (*n* = 25) were classified as compromised hosts, i.e., FRI Class R2 or R3 or BACH Class H2.

### 2.2. Diagnostic Microbiology

Overall, 19 patients were diagnosed as microbiologically positive for infection, defined as growth of phenotypically identical organisms from two or more separate deep-tissue specimens or growth of a virulent pathogen from a single specimen [12]. Monomicrobial infections were most common, identified in 16 patients (39%), with *S. aureus* as the most frequently isolated pathogen (6; 14.6%). Three polymicrobial infections were observed exclusively in patients with cOM (Table 1). Overall, 22 patients were identified as culture-negative. Among patients without prior antibiotics, 5/13 (38.5%) were culture-negative versus 17/28 (60.7%) with prior antibiotics (*p* = 0.31).

### 2.3. Diagnostic Histology

**Figure 1:**
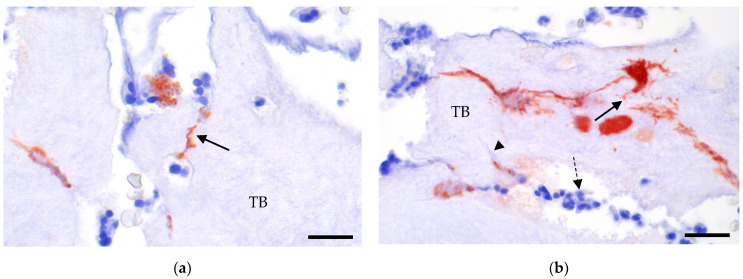

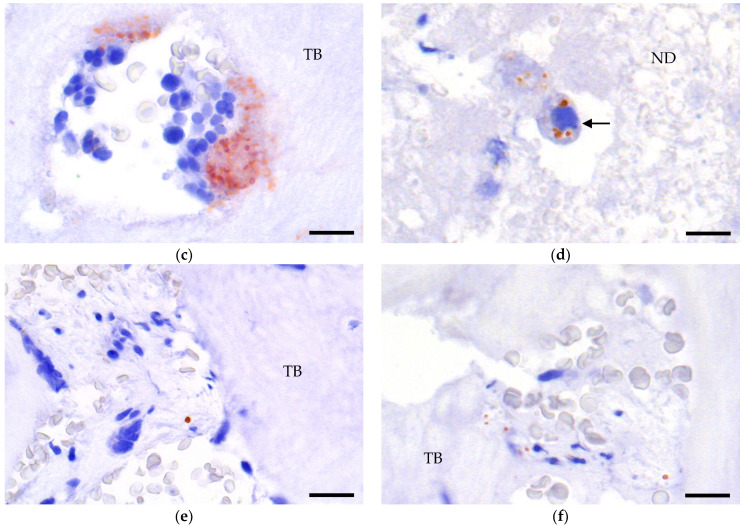
Localization of *Staphylococcus aureus* in intraoperative sampled bone biopsies from FRI patients. Immunohistochemical staining of *S. aureus*. (**a**,**b**) Infiltration of *S. aureus* (red) within the osteocyte lacuna-canalicular network (OLCN) (arrow) and microcracks of viable bone (arrowhead). Surrounding inflammatory cells (dashed arrow) are unable to reach these bacteria. (**c**) Extracellular aggregation of *S. aureus* surrounded by immune cells. (**d**) Intracellular *S. aureus* within a macrophage (arrow). (**e**,**f**) *S. aureus* observed as single isolated bacteria. Clear cells are erythrocytes. Bar; (**a**,**b**) = 100 μm, (**c**) = 40 μm, (**d**) = 10 μm, (**e**,**f**) = 150 μm. Abbreviations: TB, Trabecular bone; ND, Necrotic debris.

Among FRI patients, 26 (86.7%) were diagnosed as histologically positive for infection with a histology score of three. Two patients (6.7%) scored two and one, respectively. Among cOM patients, eight (72.7%) scored three, one (9.1%) scored two, and two (18.2%) scored one. The tissue morphology of the samples was highly heterogeneous, ranging from dense neutrophil infiltrates to sparse infiltration of inflammatory cells, and included varying degrees of granulation tissue, fibroplasia, viable bone, and necrotic bone (Figure 1). *S. aureus* were identified in all six patients with *S. aureus* positive cultures. Among the 30 samples examined (5 samples × 6 patients), 20 were culture-positive, and in 17 of these (80.9%), red/brown *S. aureus* were identified. The bacteria appeared as small intra- or extracellular colonies closely associated with inflammatory infiltrates, red blood cells, or necrotic trabecular bone (Figure 1). Furthermore, bacteria alone could be observed within the osteocyte lacuno-canalicular network (OLCN). Additionally, in six samples (from four different patients), large colonies were identified, partially surrounded by immune cells.

### 2.4. Paired Microbiology and Histology

**Figure 2:**
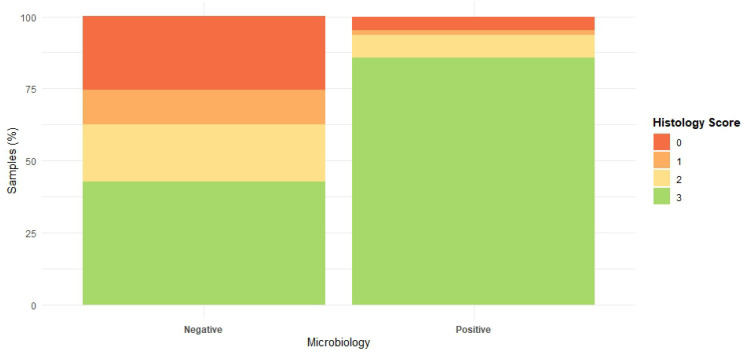
Distribution of histology scores (0–3) among culture-negative and culture-positive samples.

In total, 205 paired samples (41 patients × 5 samples each) were analysed of which 64 (31.2%) were culture-positive, 114 (55.9%) had positive histology, and 54 (26.3%) were positive by both modalities (Table 2). At the patient level, eighteen (43.9%) had at least one paired-positive sample. The number of paired positive samples per patient ranged from one to five; four patients had all five samples paired-positive, two patients had 4/5, six patients had 3/5, two patients had 2/5, and four patients had 1/5. Histological scores were significantly higher in culture-positive samples (*p* < 0.001), with scores tightly clustered around three (IQR: 3–3). In contrast, culture-negative samples had a median histology score of two with a broader distribution (IQR: 0–3) (Figure 2). Microbiology-positive samples had 8.1-fold higher odds of showing marked neutrophilic infiltration compared with culture-negative samples (95% CI 3.7–17.6).

### 2.5. Clinical Failure for Diagnostic Microbiology and Paired Positivity, Respectively

Clinical failure was more frequent in patients diagnosed as microbiology-positive (6 patients; 35%) than in patients diagnosed as culture-negative (1 patient; 5%) (*p* = 0.016). All seven failures had ≥5 NPs/HPF at index surgery. Among patients who received preoperative antibiotics (*n* = 27), clinical failure occurred in 5/10 (50%) diagnosed as microbiology-positive patients versus 1/17 (5.9%) diagnosed as culture-negative patients (*p* = 0.015). In the antibiotic-holiday subgroup, failures were rare 1/7 (14%) in microbiology-positive patients vs. 0/5 in culture-negative patients (*p* = 1.00).

**Figure 3:**
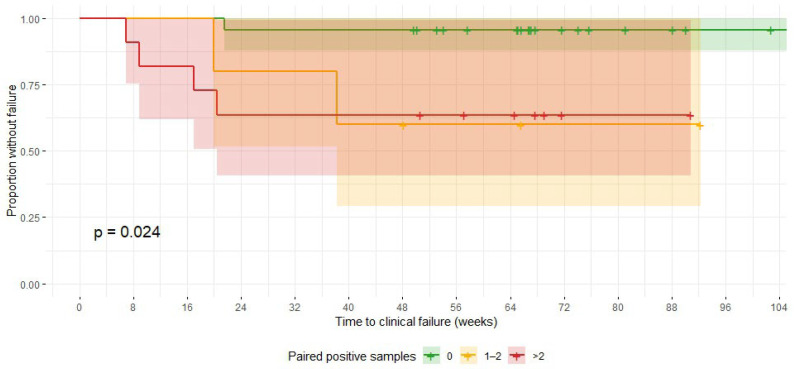
Kaplan–Meier plot showing time to clinical failure stratified by the number of paired positive tissue samples (defined as culture-positive and with a histological score of 3). Each step indicates a failure, and tick marks represent censored cases. Shaded areas represent 95% confidence intervals. Patients with ≥2 paired positive samples showed significantly higher and earlier risk of failure (log-rank *p* = 0.024).

The prognostic relevance of paired positive microbiology and histology was assessed. Patients with at least one paired positive sample, defined as a tissue specimen that was both culture-positive and showed maximal histological inflammation (score 3), had significantly increased odds of clinical failure (OR 9.3; 95% CI 1.7–98.3). The number of paired positive samples was also associated with outcome; each additional sample increased the odds of failure (OR per sample 1.68; 95% CI 1.10–2.77). Failure was rare in patients without paired positive samples whereas those with two or more samples failed earlier (Table 3, Figure 3).

### 2.6. Paired Microbiology and Osteoclast Activity

Osteoclast activity was predominantly absent, with 147/204 (72%) samples graded as 0, 48/204 (24%) graded as 1, and 9/204 (4%) graded as 2 (Figure 1). The distribution of osteoclast scores did not differ substantially between culture-negative and positive samples (*p* = 0.23, Table 2).

## 3. Discussion

The paired design of the present study identified that culture-positive samples exhibit an increased number of neutrophils on histology. Therefore, the study hypothesis regarding the inflammatory response was supported. In chronic bone infections, chemotaxis recruits NPs, leading to non-stochastic accumulation in close proximity to bacteria. An acute inflammatory response dominated by neutrophil infiltration typically resolves through processes that promote tissue repair and restore homeostasis [28]. However, the persistence of pathogens in chronic or biofilm-associated infection sustains a neutrophil-dominated response. The present IHC findings also visualized this, by showing a heterogeneous and spatial distribution of bacteria co-localized with inflammatory infiltrates.

Paired microbiological and histological positivity predict clinical failure, and multiple paired positive samples are associated with earlier revisions. Failures occur due to non-resected residual viable bacteria or recolonization after revision surgery. Regardless of the mechanism, it is well established that bacteria can evade clearance through several strategies, including intracellular invasion, colonization of OLCN, formation of small-colony variants, biofilms, and abscesses [29]. Furthermore, bone necrosis may allow bacterial persistence within avascular areas that are not resected during surgery. The relationship between the observed treatment failures and paired positivity, at the time of index surgery, might be affected by the host’s inflammatory response, where neutrophils serve as the primary effector cell, driving bone tissue destruction and fibrosis formation (although the severity of these responses can be highly pathogen dependent). It is plausible that an intensified or heightened inflammatory response facilitates bacterial ingress and dissemination beyond the initially infected zone. Moreover, the presence of intracellular bacteria is noteworthy and aligns with findings from other recent studies, highlighting its clinical relevance [30]. A higher inflammatory state (both culture- and histology-positive) may reflect an abundance of inflammatory cells susceptible to becoming “Trojan horse” cells. These cells can support bacterial evasion of both immune- and antibiotic-mediated clearance, thereby contributing to clinical failure. Consequently, a paired positivity signals an active inflammatory response that may promote various bacterial evasion strategies, increasing the risk of a residual bacterial burden.

A high proportion of culture-negative cases was observed, especially among those who received preoperative antibiotics (Table 2). The proportion of culture-negative patients was higher than in most recent reported FRI and cOM series (8–18%) [31,32,33]. Preoperative antibiotics have been described to influence culture sensitivity along with the number and location of collected samples—particularly at the bone–implant interface and within necrotic bone [2,7]. To obtain a reliable culture-result it is recommended to aseptically collect at least five separate deep tissue samples and withhold systemic antibiotics for at least two weeks before surgery to reduce false-negative results [1,11,34]. High antibiotic tissue concentrations can delay detection or completely prevent bacterial growth, resulting in apparently sterile cultures despite ongoing infection [9,10].

The high proportion of culture-negative, yet histology-positive cases observed in this study, further supports a complementary paired diagnostic strategy. Morgenstern et al. demonstrated that quantitative histological assessment improves the diagnostic accuracy of FRIs, particularly in cases with negative or inconclusive microbiological results [12]. A previous study demonstrated that the neutrophil infiltration is most pronounced centrally within the infection sites and decreases outwards, still leaving a mild response after debridement [18]. This “leftover” mild inflammation might be highly valuable for promoting bone healing after the debridement procedure.

Despite the reported success of diagnostic histology, it has historically been underused and inconsistently applied regarding optimal sampling. Many published articles examined only one to three histology specimens [1,17,35]. In periprosthetic joint infection, Sigmund et al. showed that submitting 3–6 histology specimens maximize diagnostic performance, supporting multi-site sampling rather than one or two samples [36]. Histology was not included in the original FRI definition [37], as no evidence was available at that time, but the revised consensus now incorporates histology [2]. Current evidence supports obtaining multiple, anatomically targeted, site-matched histology specimens and routinely including histology in secondary fracture surgery when infection is suspected [17]. Although formal cost-effectiveness analyses comparing paired microbiology and histology with microbiology alone are lacking, several studies have demonstrated that the dual approach substantially improves diagnostic accuracy and is therefore considered the diagnostic “gold standard” [12,38,39]. Considering that the cost of histological assessment is low relative to the clinical and economic burden of missed infection, this approach may be particularly valuable in cases managed with antibiotic suppression, where culture yield could be reduced and the risk of false-negative results are high.

To gain more information from the applied paired microbiology and histology set-up, osteoclast activity was also examined. Experimental work has demonstrated that bacteria, especially *S. aureus*, can directly and indirectly promote osteoclast differentiation and bone resorption. In vitro studies have shown that Gram-positive bacterial components such as Protein A, peptidoglycan, and lipoproteins activate osteoclast precursors via NF-κB signalling, while in vivo animal models confirm enhanced osteoclastogenesis and osteolysis during *S. aureus* infection [40]. Similarly, a recent review highlighted that different bacteria exert heterogeneous effects on osteoclasts, with *S. aureus* being strongly osteoclastogenic, whereas organisms such as *S. epidermidis* induce only mild inflammation and little bone destruction [41]. In contrast, the present human FRI and cOM cohorts did not show increased osteoclast activity in culture-positive compared with culture-negative samples, suggesting that in chronic, and often antimicrobial pretreated infections, osteolysis may be less dependent on bacterial viability and osteoclasts than on the inflammatory response per se.

### 3.1. Limitations

The study lacked a control group with non-paired sampling, as histology is not routinely performed at the current centre. The small cohort size and single-centre design weaken the prognostic values of the proposed thresholds. This is reflected in the wide confidence intervals, and the results should therefore be interpreted with caution. Pooling FRI and cOM patients may be a limitation, as their biological and clinical presentations differ; however, they share similar underlying pathophysiological features of bone infection, which justified combining them in this exploratory cohort. Also, there are no studies which advocate different microbiological or histological diagnostic criteria for these conditions. Larger, multicentre studies with increased sample sizes are needed to validate these thresholds. Furthermore, splitting the samples, for parallel microbiological and histological assessment may introduce error as bacterial load and neutrophil infiltration may vary even within small regions of infected tissue [16]. The proportion of culture-negative cases was high, and molecular diagnostics could have further clarified culture-negative results.

### 3.2. Conclusions

Culture-positive samples showed increased neutrophil infiltration without a corresponding rise in osteoclast activity. Paired positivity between culture and histology was associated with earlier and more frequent failures. Although this is a small study and should be interpreted accordingly, the results present an interesting and novel observation, namely that combining modalities on paired samples may help reveal disease severity and provide prognostic value. However, the observation requires further clinical validation and can currently serve only as a solid foundation for more extensive research on the topic. Finally, adding histology to the diagnostic protocol proved beneficial, particularly in culture-negative cases.

## 4. Materials and Methods

### 4.1. Patient Cohort

**Figure 4:**
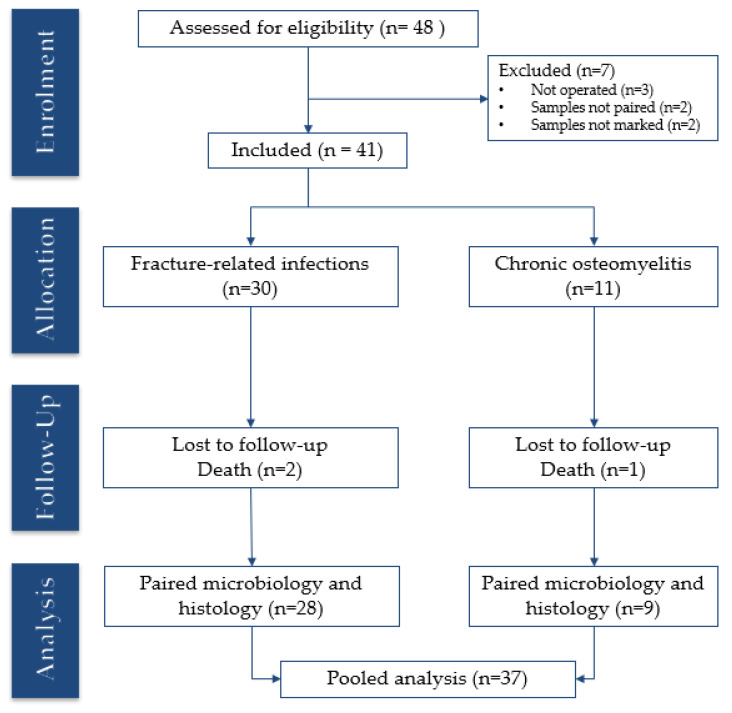
CONSORT flow diagram of the study cohort. Illustrates patient screening, inclusion, allocation into fracture-related infection and chronic osteomyelitis groups, losses to follow-up, and final numbers included in the paired analysis.

The study was conducted in a single-centre cohort at a tertiary orthopaedic infection centre, where patients were prospectively enrolled by a specialized multidisciplinary team at Herlev Hospital, Capital Region, Denmark. Patients were included between May 2023 and August 2024 (Figure 4), with a minimum follow-up of 12 months. Inclusion criteria were age ≥18 years and undergoing surgery for FRI or cOM. FRI was diagnosed using the confirmatory criteria of the International Consensus Definition [2,34]. cOM was defined as an infection persisting for more than six weeks, with a draining wound or sinus tract communicating with dead bone and with local and/or systemic features of inflammation [38,42]. Microbiology and histology were excluded from the diagnostic criteria. Clinical confirmation included the presence of a fistula, sinus tract, wound breakdown, or abscess/pus around the fracture or implant, or within the affected bone in cases of cOM. Contrast-enhanced magnetic resonance imaging was performed when indicated.

Clinical data were collected for each patient, including age, sex, body mass index, and anatomical localization of infection. Classification of bone infection was based on either the FRI classification, which assesses three key components; (1) Fracture (F), (2) Related patient factors (R), and (3) Impairment of soft tissues (I), or the BACH classification for cOM, which evaluates Bone involvement (B), Antimicrobial options (A), soft-tissue Coverage (C), and Host status (H) [26,27]. Other demographic and clinical variables, including diabetes mellitus, heart failure, renal failure, liver disease, obesity (BMI ≥ 30), active malignancy, smoking, and substance misuse were recorded alongside treatment details and clinical outcomes. Preoperative antibiotic cessation was documented, with an antibiotic-free interval of at least two weeks considered an antibiotic holiday. The minimum follow-up period was 12 months. Clinical failure was defined as the need for revision surgery within this period, including debridement for a non-healing wound, a persistent sinus tract, wound breakdown, reoccurrence of fistula, or any proximal amputation.

### 4.2. Surgery and Tissue Sampling

**Figure 5:**
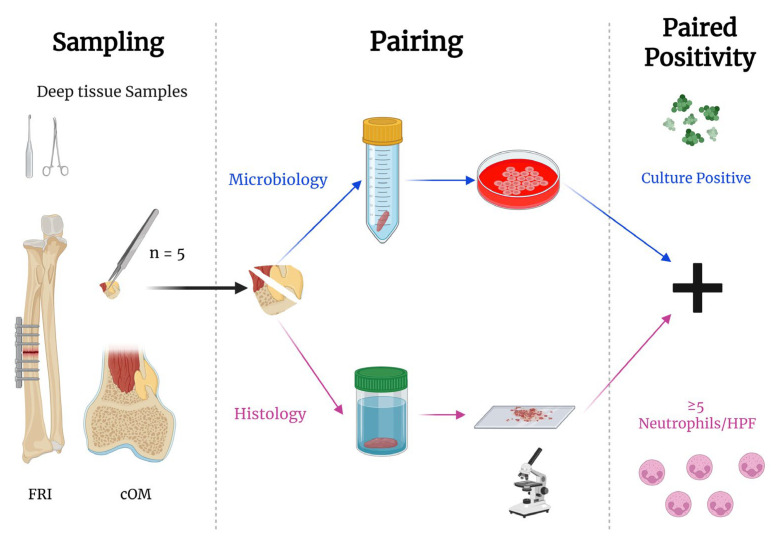
Schematic of tissue sampling in a fracture-related infection case. Five deep-tissue samples were collected at the start of surgery using separate sterile instruments. Each sample was split aseptically into two matched halves: one half was submitted for microbiology (sterile container for culture), and the other was fixed in formalin and processed/sectioned for histopathology. Abbreviations: FRI, Fracture-related infections; cOM, chronic osteomyelitis. Created with BioRender.com.

During the index surgery, any fistula, scar tissue, or necrotic skin and subcutaneous tissue were excised. As early as possible, five deep-tissue specimens were collected and aseptically divided into paired samples for parallel microbiological and histological evaluation (Figure 5). Each specimen was obtained with a separate set of sterile instruments. In FRI cases, all metal implants and other foreign material (e.g., sutures, pins) were removed when feasible. In cases managed with debridement, antibiotics, and implant retention (DAIR) or with implant exchange, the hardware was retained or replaced only after thorough debridement. For both cOM and FRI cases, all infected bone and soft tissue were meticulously excised. The surgical cavity was irrigated with isotonic saline, and residual bone voids were filled under short tourniquet control using an antibiotic-eluting calcium sulphate–hydroxyapatite graft (CERAMENT G, containing 17.5 mg/mL gentamicin; BONESUPPORT AB, Lund, Sweden). Wounds were closed primarily when tension-free closure was possible; otherwise, soft-tissue coverage was achieved with a local musculocutaneous flap in collaboration with a plastic surgeon.

Postoperatively, empirical intravenous antibiotic therapy with dicloxacillin (1000 mg) and penicillin (1200 mg) was administered every six hours. The antibiotic regimen was subsequently tailored based on culture and sensitivity results and converted to oral therapy within seven days. A total of six weeks of systemic antibiotic therapy was administered, following the OVIVA trial recommendations [19], and extended to 12 weeks in cases where metal implants were retained at index surgery.

### 4.3. Microbiological Analysis

Microbiological assessment included inoculation of tissue samples on 5% Columbia blood agar plates and on anaerobe agar plates (Media Preparation Laboratory, Herlev, Denmark) for aerobic (Atmospheric air, 5% CO_2_) and anaerobic cultures, respectively. Incubation time was 5 days. Additionally, specimens were cultured thioglycolate enrichment broth (Media Preparation Laboratory, Herlev, Denmark) for 14 days at 35 °C (Atmospheric air, 5% CO_2_). Pathogen identification was performed using MALDI-TOF mass spectrometry (Bruker Daltonics GmbH & Co. KG, Bremen, Germany) using MBT Compass HT software (Version 5.4.420.61), and MBT Compass library 2023 according to the manufacturer’s protocols. Antimicrobial susceptibility testing followed EUCAST guidelines [43]. Sample was considered microbiologically positive in case of a positive culture. Microbiological diagnostic confirmation of infection in the individual patient was defined by isolating phenotypically indistinguishable pathogens from at least 2/5 separate deep tissue specimens [37]. Single diagnostic positive cultures (1/5) could only confirm infection in the individual patient when a virulent pathogen was isolated. Virulent pathogens included Gram-negative bacilli, *Staphylococcus aureus*, *Staphylococcus lugdunensis*, enterococci, beta-haemolytic streptococci, *Streptococcus pneumoniae*, milleri group streptococci, and *Candida* species [33]. Cases were only classified as culture-negative after extended incubation for 14 days without pathogen growth.

### 4.4. Histopathological Analysis

Bone specimens were fixed in formalin using alternating cycles of vacuum (−0.1 MPa for 2 h) and atmospheric pressure (2 h) over a 48-h period. Following fixation, the samples underwent four weeks of decalcification in ethylenediaminetetraacetic acid (EDTA; Sigma-Aldrich, St. Louis, MO, USA). After decalcification, all samples were trimmed, processed through graded alcohols and xylene, embedded in paraffin wax and sectioned to a thickness of 4–5 μm. All sections were stained with haematoxylin and eosin (H&E) for histological evaluation.

All tissue sections were examined to identify areas with the highest concentration of NPs. At least ten HPFs at ×400 magnification were evaluated in each inflamed area. Neutrophil infiltration was graded in four categories (0–3): 0 = no NPs; 1 = an average of fewer than one NP per HPF; 2 = an average of one to five NPs per HPF; and 3 = an average of more than five NPs per HPF. This scoring system was applied consistently across all cases. Histological evaluation was performed independently by an Orthopedic resident (A.A.N.P.) and an experienced pathologist (L.K.J), both blinded to clinical and microbiological data. A sample was considered histologically positive if it received a score of 3 [12,33,37]. Osteoclasts were semi-quantitatively graded on a 0–3 scale across 10 HPFs, where 0 = none, 1 = <1 osteoclast/HPF, 2 = 1–3 osteoclasts/HPF, and 3 = >3 osteoclasts/HPF.

### 4.5. Immunohistochemistry for S. aureus

Samples positive for *S. aureus* by microbiological culture were selected for immunohistochemical (IHC) staining towards *S. aureus*. Paraffin-embedded tissue sections (4–5 µm) were mounted on adhesive glass slides (Thermo Scientific, Menzel GmbH & CoKG, Braunschweig, Germany), dewaxed, and rehydrated. Endogenous peroxidase activity was blocked with 0.6% hydrogen peroxide, diluted in tris-buffered saline (TBS), for 15 min, followed by blocking with 2.5% normal goat serum for 5 min. Sections were incubated overnight at 4 °C with a rabbit polyclonal anti–*S. aureus* antibody (PA1-7246; Invitrogen, Toulouse, France) diluted 1:38,400 in TBS containing 1% bovine serum albumin. Detection was performed using the ImmPRESS HRP Goat Anti-Rabbit IgG Polymer Kit (MP-7451; Vector Laboratories, Newark, CA, USA), and signal was visualized with aminoethyl carbazole chromogen (SK-4200; Vector Laboratories) according to the manufacturer’s protocol. Finally, slides were counterstained with Mayer’s haematoxylin and mounted with glycerol-gelatine. Throughout the immunostaining protocol, tissue sections were washed in TBS (pH 7.6). Positive controls were performed on *S. aureus*–infected porcine lung tissue, while negative controls included omission of the primary antibody or substitution with rabbit IgG (DAKO X0903, Merck, Darmstadt, Germany). Red/brown bacterial colonies were classified as positive, and both their location and size (small or large) were registered. The distinction between small and large bacterial colonies was based on their size in relation to neutrophil granulocytes. A bacterial colony with a diameter greater than the size of two NPs (~24 µm) was considered large.

### 4.6. Statistical Analysis

Baseline demographic and clinical characteristics were summarized using medians with quartiles for continuous variables and counts with percentages for categorical variables. Comparisons between FRI and cOM groups were performed using the Mann–Whitney U test for continuous variables and Fisher’s exact test for categorical variables. In subsequent analyses, cOM and FRI samples were pooled to increase statistical power and because of their shared diagnostic principles; thus, they were not examined separately.

Differences in histological neutrophil infiltration scores (0–3) between culture-positive and -negative samples were evaluated using a Mann–Whitney U test. To further examine this relationship, an ordinal logistic regression model was fitted with microbiological status as the predictor. Patients were classified as ‘paired positive’ if ≥1 tissue sample was both culture-positive and had a histology score of 3. Associations with 12-month clinical failure were examined using Firth logistic regression. Only covariates with a univariate *p* < 0.05 were carried forward to multivariable analysis. To reduce sparsity, BACH/FRI and microbiology variables were collapsed as follows: host (H1/R1 vs. H2/R2/R3), bone (B1/F1 + F2 [Optimal] vs. F3 [Suboptimal]), soft tissue (C1/I1/I2 vs. C2/I3), and microbiology (culture-negative vs. monomicrobial/polymicrobial). Patients who died within the 12-month follow-up without evidence of failure were excluded from outcome analysis. Analyses were performed R version 4.2.3 (R Foundation for Statistical Computing, Vienna, Austria); two-sided *p* < 0.05 was considered statistically significant.

## Figures and Tables

**Table 1:** Baseline characteristics and outcomes of cOM and FRI patients.

Variable	Overall *n* = 41	cOM *n* = 11	FRI *n* = 30	*p*-Value
Age, y	63 (44–73)	71 (61–82)	58.5 (33.5–72)	0.037
BMI, kg/m^2^	24.9 (22.2–29.1)	25.3 (23.3–29.3)	27.1 (6.4)	0.12
Male (%)	20 (48.8)	8 (72.7)	12 (40)	0.085
Comorbidities (%)				
Old age (>80 y)	7 (17.1)	4 (36.4)	3 (10.0)	0.069
Diabetes	4 (9.8)	0	4 (13.3)	0.56
Heart failure	8 (19.5)	4 (36.4)	4 (13.3)	0.18
COPD	3 (7.3)	2 (18.2)	1 (3.3)	0.17
Renal failure	3 (7.3)	1 (9.1)	2 (6.7)	1.0
Liver disease	2 (4.9)	0	2 (6.7)	1.0
Obesity (BMI > 30)	7 (17.1)	1 (9.1)	6 (20)	0.65
Active cancer	1 (2.4)	1 (9.1)	0	0.27
Smoker	19 (46.3)	6 (54.5)	13 (43.3)	0.73
Drug misuse	7 (17.1)	1 (9.1)	6 (20.7)	0.65
Peripheral arterial disease	3 (7.3)	2 (18.2)	1 (3.3)	0.17
Pre-op antibiotics	28 (68.3)	7 (63.6)	21 (70)	0.72
Classification (%)				
BACH	11 (26.8)			
B1		11 (100)		
A1		5 (45.5)		
Ax		5 (45.5)		
A2		1 (9.1)		
C1		10 (90.9)		
C2		1 (9.1)		
H1		4 (36.4)		
H2		7 (63.6)		
Uncomplicated		4 (36.4)		
Complex		7 (63.6)		
FRI *	30 (73.2)			
F1			17 (56.7)	
F2			7 (23.3)	
F3			6 (20)	
R1			12 (40)	
R2			13 (43.3)	
R3			5 (16.7)	
I1			18 (60)	
I2			9 (30)	
I3			3 (10)	
Microbiology groups (%)				
*S. aureus*	6 (14.6)	0	6 (20)	
Other CoNS	7 (17.1)	3 (27.3)	4 (13.3)	
Gram-negative Bacilli	1 (2.4)	0	1 (3.3)	
Other Gram-positive	2 (4.9)	0	2 (6.7)	
Polymicrobial	3 (7.3)	3 (27.3)	0	
Culture-Negative	22 (53.7)	5 (45.5)	17 (56.7)	
Outcome ^#^				
Success	32 (82.1)	10 (100)	22 (75.9)	
Death (<12 months)	2 (4.9)	1 (9.1)	1 (3.3)	
Revised	7 (17.9)	0	7 (24.1)	

Medians (Q1–Q3) are shown for continuous variables, counts and n (%) for categorical. Abbreviations: cOM, chronic osteomyelitis; FRI, fracture-related infections; y, years; B, bone; A, antimicrobial options; C, coverage by soft tissue; H, host; F, fracture; R, related patient factors; I, impairment of soft tissues [26,27]. CoNS, Coagulase-negative staphylococci. * FRI classification has no overall stratification. ^#^ Two patients who died within the 12-month follow-up period without evidence of failure were excluded from this analysis.

**Table 2:** Distribution of microbiology-positive samples by histology and osteoclast scores.

Category	Score	Microbiology-Negative, *n* (%)	Microbiology-Positive, *n* (%)	Total
Histology	0	36 (92.3)	3 (7.7)	39 (19.1)
	1	17 (94.4)	1 (5.6)	18 (8.8)
	2	28 (84.8)	5 (15.2)	33 (16.2)
	3	60 (52.6)	54 (47.4)	114 (55.9)
Osteoclasts	0	105 (71.4)	42 (28.6)	147 (72.1)
	1	31 (64.6)	17 (35.8)	48 (23.5)
	2	5 (55.6)	4 (44.4)	9 (4.4)
Total		141 (69.1)	63 (30.9)	204 *

Counts and *n* (%). * 1 sample was missing for histological evaluation.

**Table 3:** Association between number of matched pairs and risk of clinical failure.

Number of Matched Pairs	Clinical Failure *n* (%)	Odds Ratio (95% CI)	Time to Failure Weeks (IQR)
0	1/23 (4.3%)	reference	22
1–2	2/5 (40%)	10.7 (1.11–148.6)	29 (25–34)
≥3	4/11 (36.4%)	9 (1.38–101.3)	13 (8–18)

Abbreviations: CI, confidence interval; IQR, interquartile range.

## Data Availability

The data supporting the findings of this study are available from the authors upon request and without undue reservation.
